# Association between meat, fish, and fatty acid intake and incidence of acute myeloid leukemia and myelodysplastic syndrome: the Japan Public Health Center-based Prospective Study

**DOI:** 10.1265/ehpm.22-00233

**Published:** 2023-03-07

**Authors:** Yoshimitsu Shimomura, Tomotaka Sobue, Ling Zha, Tetsuhisa Kitamura, Motoki Iwasaki, Manami Inoue, Taiki Yamaji, Shoichiro Tsugane, Norie Sawada

**Affiliations:** 1 Department of Environmental Medicine and Population Science, Graduate School of Medicine, Osaka University, Suita, Osaka, Japan; 2 Department of Hematology, Kobe City Hospital Organization Kobe City Medical Center General Hospital, Kobe, Japan; 3 Division of Epidemiology, National Cancer Center Institute for Cancer Control, Tokyo, Japan; 4 Division of Prevention, National Cancer Center Institute for Cancer Control, Tokyo, Japan; 5 Division of Cohort Research, National Cancer Center Institute for Cancer Control, Tokyo, Japan; 6 National Institute of Health and Nutrition, National Institutes of Biomedical Innovation, Health and Nutrition, Tokyo, Japan

**Keywords:** Acute myeloid leukemia, Incidence, Japan public health center–based study, Myelodysplastic syndrome, Processed red meat

## Abstract

**Background:**

The association between meat, fish, or fatty acid intake and acute myeloid leukemia (AML) and myelodysplastic syndrome (MDS) has been investigated in a few studies, and the results were inconsistent. In addition, most studies are mainly based on the United States and European countries, in which the dietary patterns differ from that in Asia. Therefore, the risk of AML/MDS from meat, fish, or fatty acid intake in Asia requires further exploration. The aim of this study was to investigate the association between AML/MDS incidence and meat, fish, or fatty acid intake using the Japan Public Health Center–based prospective study.

**Methods:**

The present study included 93,366 participants who were eligible for analysis and followed up from the 5-year survey date until December 2012. We estimated the impact of their intake on AML/MDS incidence using a Cox proportional hazards model.

**Results:**

The study participants were followed up for 1,345,002 person-years. During the follow-up period, we identified 67 AML and 49 MDS cases. An increased intake of processed red meat was significantly associated with the incidence of AML/MDS, with a hazard ratio of 1.63 (95% confidence interval, 1.03–2.57) for the highest versus lowest tertile and a P_trend_ of 0.04. Meanwhile, the intake of other foods and fatty acids was not associated with AML/MDS.

**Conclusion:**

In this Japanese population, processed red meat was associated with an increased incidence of AML/MDS.

**Supplementary information:**

The online version contains supplementary material available at https://doi.org/10.1265/ehpm.22-00233.

## Background

Acute myeloid leukemia (AML) represent a genetically heterogeneous group of myeloid neoplasms that primarily affect older adults [1]. Myelodysplastic syndromes (MDS) shares a part of clinical, pathological feature with AML, and is distinguished from AML by lower percentage of blast [2]. An accurate understanding of the etiology of both diseases is essential for their prevention because they have high morbidity and mortality [1, 2]. Although the etiologies of AML/MDS are poorly understood, exposure to chemicals such as benzene, dioxin, pesticides, and herbicides is associated with the development of AML or MDS [1, 2]. Benzene is the most consistently-identified leukemogenic chemical and implicated in the development of AML and MDS via benzene-related oxidative stress and aryl hydrocarbon receptor dysregulation [3, 4]. Some studies revealed the possible association between dioxin exposure and AML development [5], pesticide exposure and AML [6] or MDS [7] development, and herbicides and AML [6] or MDS [8] development. Among the potential causes of diseases, food intakes may be related to the incidence of AML/MDS. For example, red meat and processed red meat intakes can increase the incidence of AML/MDS similar to other cancers due to carcinogens, such as polycyclic aromatic hydrocarbons and N-nitroso compounds [9–11]. Similarly, fish intakes, which is generally associated with a reduced risk of cancer, can increase the incidence of AML/MDS [12] due to its components, including sea pollution such as heavy metals [13] and dioxin [14, 15]. In addition, fatty acids, an important component of meat and fish, are associated with other hematological malignancies and are possibly associated with AML/MDS risk [16–19]. However, most epidemiological studies regarding association between meat and fish intakes and AML/MDS incidence are case-control studies, which induce publication bias and recall bias, there are a few good cohort studies with less bias, and the reported results were inconsistent [18, 20–29]. In addition, most studies were performed in the United States and European countries, in which the dietary patterns differed from that in Asia [12, 18, 20–29]. Therefore, the risk of AML/MDS from meat, fish, or fatty acid intake in Asia requires further exploration.

Thus, this study aimed to investigate the association between meat, fish, or fatty acid intake and the incidence of AML/MDS using Japan public health center (JPHC)–based prospective study data.

## Materials and methods

### Study population

The present study included 93,366 participants. All participants were registered in the JPHC. The details of the JPHC study are described elsewhere [30]. Briefly, the JPHC study comprised cohorts I and II, which started in 1990 and 1993, respectively. The cohort included 11 public health centers with a total of 140,420 inhabitants across Japan.

This study excluded participants from Katsushika, Tokyo, who had no cancer incidence data (n = 7,097), who were not Japanese (n = 52), who emigrated before the start of the study (n = 188), whose recorded birth date was incorrect (n = 7), for whom registration data were duplicated (n = 12), who emigrated the original area before the 5-year survey (n = 5,060), who died before the 5-year survey (n = 6,598), and who were lost to follow-up before the 5-year survey (n = 73). Among remaining 121,333 participants, 98,616 participants returned the 5-year follow-up questionnaire, which yielded a response rate of 81.3%. We excluded the participants who did not participate in the 5-year follow-up questionnaire used as an exposure assessment because we used a more inclusive food frequency questionnaire (FFQ) in the 5-year follow-up questionnaire. Furthermore, we excluded participants who had a previous cancer history (n = 2,943, 3.0%), were lost to follow-up (n = 214, 0.2%), or reported an intake of over 5000 kcal/day or less than 500 kcal/day (n = 2,093, 2.1%). The study was approved by the ethics committees of the National Cancer Center (Approval number: 2015-085, date: November 25, 2021) and Osaka University (Approval number: 14020-10, date: February 10, 2021).

### Exposure assessment

Daily food consumption (g/day) was calculated by multiplying the daily consumption frequency by the selected portion size using a FFQ obtained at the 5-year survey. The FFQ included 138 food and beverage items with standard portions/units (small, medium, and large portions) and nine frequency categories (almost never, 1–3 times/month, 1–2 times/week, 3–4 times/week, 5–6 times/week, 1 time/day, 2–3 times/day, 4–6 times/day, and ≥7 times/day). The average consumption of 16 meat items and 19 fish and shellfish items during the previous year was calculated from the standard portion and frequency categories obtained from the FFQ. We categorized these food items into the following groups: total meat including all meat items; red meat including all 10 unprocessed red meat items and all 4 processed red meat items; unprocessed red meat including steak, grilled beef, and stewed beef, stir-fried pork, deep-fried pork, Western-style stewed pork, Japanese-style stewed pork, pork in soup, and pork liver, and chicken liver, processed red meat including ham, sausage or Western-style sausage, bacon, and luncheon meat, poultry including grilled chicken and deep-fried chicken, fish including salted fish, dried fish, canned tuna, salmon or trout, bonito or tuna, codfish or flatfish, sea bream, horse mackerel or sardines, mackerel pike or mackerel, dried small fish, salted roe, eel, squid, octopus, prawns, short-necked clam, viviparidae, chikuwa, and kamaboko, big fish including salmon, skipjack/tuna, cod/flatfish, and sea bream, and small fish including horse mackerel/sardine, saury/mackerel, and eel [31].

In addition to meat and fish intake, the dietary intake of total fatty acid, saturated fatty acid, monounsaturated fatty acid, polyunsaturated fatty acid (PUFA), n-3 PUFA, and n-6 PUFA were calculated according to the fatty acid composition table developed by the substitute method based on fatty acid composition tables for Japanese foods [32]. The intake of meat, fish, and fatty acids was adjusted for total energy intake by the residual model [33].

The validity of the FFQ was evaluated using energy-adjusted Spearman’s correlation coefficients between the intake of interest derived from the FFQ and those derived from the 28-day or 14-day dietary records [34, 35]. The energy-adjusted Spearman’s correlation coefficients of meat were 0.50 for men and 0.47 for women, those of fish were 0.32 for men and 0.32 for women, and those of fatty acid were 0.52 for men and 0.47 for women [34, 35]. The reproducibility of the FFQ was evaluated by energy-adjusted Spearman’s correlation coefficients for the intake of interest derived from the two FFQ administered 1 year apart [36, 37]. The energy-adjusted Spearman’s correlation coefficients of meat were 0.52 for men and 0.52 for women, that of fish were 0.44 for men and 0.34 for women, and that of fatty acid were 0.47 for men and 0.52 for women [36, 37].

### Case identification

Newly diagnosed AML/MDS cases were determined from medical reports of major local hospitals and data linkage with population-based cancer registries. Death certificate information was used as a supplementary information source. Data linked between major local hospitals and population-based cancer registries were used to confirm newly diagnosed AML/MDS. The cases were coded according to the hospital-based cancer registries in Japan using the International Classification of Diseases for Oncology, Third Edition. The histologic codes were 9840, 9860, 9861, 9866, 9867, 9873, 9874, 9875, 9891, 9895, and 9896 for AML and 9980, 9982, 9983, 9985, and 9987–9989 for MDS. If two or more AML/MDS or other cancers were diagnosed in one participant, the first diagnosis was used in the analysis.

### Endpoints and statistical analysis

The person-years of follow-up were counted for individual participants from the 5-year follow-up survey date to the end of follow-up, which was defined as move outside the study area, loss to follow-up, withdrawal from the study, death, diagnosis of AML/MDS, or last date of the follow-up period (December 31, 2012).

Continuous variables are summarized as median and interquartile range (quartiles 1–3), while categorical variables are summarized as count and percentage. The study participants were subdivided into tertiles with respect to their energy-adjusted intakes of interest, including total meat, red meat, processed red meat, unprocessed meat, poultry, fish, total fatty acids, saturated fatty acids, monounsaturated fatty acids, PUFA, n-3 PUFAs, and n-6 PUFA. We estimated the impact of exposure on AML/MDS incidence using a multivariable Cox proportional hazard model. We described the hazard ratios (HRs), 95% confidence intervals (CIs), and P_trend_ values to test for a linear trend across tertiles as a rank variable. Adjusted covariates were associated with the incidence of AML/MDS. Information on covariates was collected from a 5-year follow-up questionnaire survey. Body mass index (BMI) was calculated as body weight (kg) divided by squared height (m^2^) and categorized as <23, 23–<25, 25–<27, and ≥27 kg/m^2^. We assessed and categorized smoking history into never smokers, past smokers, current smokers, and unknown. Alcohol consumption was assessed as the weekly consumption obtained by multiplying the weekly frequency and categorized as never, rarely, 1–3 times/month, 1–2 times/week, 3–4 times/week, >4 times/week, and unknown. Physical activity is expressed as metabolic equivalents/day and categorized as quartiles and unknown.

In model 1, adjusted covariates were age (continuous), sex, and study area (10 public health center areas). In model 2 (primary result), adjusted covariates were age (continuous), sex, study area (10 public health center area), BMI, history of smoking, alcohol consumption frequency, and physical activity as metabolic equivalents/day. Covariates were selected based on previous studies [38, 39]. In model 3, the sensitivity analyses excluded participants diagnosed with AML/MDS in the first two years to remove the potential bias of having AML/MDS at the 5-year survey which is the start of follow-up (9 cases diagnosed before 5-year survey were excluded in the analysis). Stratified analyses were conducted by estimating the interaction between intake of interest and age (≤median and >median, the median age was 57 years) and sex. Similarly, we estimated the impact of exposure on AML/MDS incidence using the same methods mentioned above.

All P values presented were two-sided, and values <0.05 were considered statistically significant. All statistical analyses were performed using Stata version 14 (StataCorp LLC, the United States).

## Results

We showed the baseline characteristics divided by the tertiles of total meat intake in Table 1. The median age was 57 (interquartile range, 51–63) years, and 46.5% of the participants were men. A total of 93,366 participants were included and followed up for 1,345,002 person-years. During the follow-up period, we identified 67 AML and 49 MDS cases.

**Table 1:** Participant Baseline Characteristics according to the Japan public health center based prospective study cohort

	**Tertile of energy adjusted total meat intake**		

	**Tertile 1**	**Tertile 2**	**Tertile 3**
Age^a^, year	58 (52–64)	56 (50–63)	56 (49–62)
Men, %	49.8	47.4	42.4
Body mass index^a^, kg/m^2^	23.2 (21.4–25.2)	23.3 (21.5–25.3)	23.5 (21.5–25.6)
Current smoker, %	24.2	24	21.5
Regular drinker, %	40.5	39.5	32.1
Metabolic equivalents^a^	31.9 (27.1–36.1)	31.9 (27.1–35.5)	31.9 (27.1–35.5)

**Intakes**			

**Food**			

Total meat^a^, g/day	22.8 (13.7–30.0)	49.8 (43.1–57.1)	89.0 (75.3–112.2)
Red meat^a^, g/day	18.1 (10.3–24.7)	41.7 (35.5–48.7)	76.4 (64.1–97.9)
Processed red meat^a^, g/day	1.5 (0.4–3.5)	4.2 (1.9–7.8)	7.2 (3.3–13.7)
Unprocessed red meat^a^, g/day	15.3 (8.3–21.6)	36.1 (29.8–43.1)	67.1 (54.9–87.2)
Poultry^a^, g/day	3.3 (0.6–6.0)	7.3 (4.2–11.1)	11.0 (6.1–18.1)
Fish^a^, g/day	68.9 (42.1–107)	79.0 (54.4–111)	79.6 (53.9–113)
Big fish^a^, g/day	14.2 (6.5–26.6)	17.5 (10.1–29.1)	19.0 (10.8–30.8)
Small fish^a^, g/day	14.2 (6.5–26.6)	17.5 (10.1–29.1)	19.0 (10.8–30.8)

**Nutrient**			

Total energy^a^, kcal/day	1912 (1464–2479)	1914 (1540–2377)	1890 (1537–2320)
Total FA^a^, g/day	37.1 (28.8–46.0)	45.3 (38.2–52.6)	56.6 (48.9–64.6)
Saturated FA^a^, g/day	12.2 (8.8–16.1)	15.3 (12.3–18.6)	19.6 (16.4–23.0)
MUFA^a^, g/day	13.8 (10.7–17.1)	17.7 (15.1–20.5)	23.1 (20.0–26.6)
PUFA^a^, g/day	10.4 (8.1–12.9)	11.7 (9.8–13.8)	13.5 (11.5–15.6)
n-3 PUFA^a^, g/day	2.1 (1.6–2.8)	2.3 (1.9–2.9)	2.5 (2.0–3.0)
n-6 PUFA^a^, g/day	8.1 (6.4–10.1)	9.3 (7.8–11.0)	10.9 (9.3–12.6)

FA: fatty acid, MUFA; monounsaturated fatty acid, and PUFA; polyunsaturated fatty acid

^a^Continuous variables are summarized as median and interquartile ranges (quartiles 1–3)

The adjusted HR, 95% CI, and P_trend_ of the incidence of AML/MDS according to the intake of dietary factors of interest were shown in Table 2. An increased intake of processed red meat was significantly associated with the incidence of AML/MDS, with an HR of 1.63 (95% CI, 1.03–2.57) for the highest versus lowest tertile and P_trend_ of 0.041. Meanwhile, red meat (HR [highest vs lowest]: 1.35, 95%CI: 0.85–2.14), unprocessed meat (HR: 1.08, 95%CI: 0.68–1.71), poultry (HR: 1.35, 95%CI: 0.85–2.13), fish (HR: 1.13, 95%CI: 0.71–1.79), big fish (HR: 1.03, 95%CI: 0.64–1.67) and small fish (HR: 1.32, 95%CI: 0.82–2.14) intake were not significantly associated with the incidence of AML/MDS. The intakes of total fatty acid (HR: 1.31, 95%CI: 0.81–1.57), saturated fatty acid (HR: 1.01, 95%CI: 0.62–1.63), monounsaturated fatty acid (HR: 1.03, 95%CI: 0.63–1.69), PUFA (HR: 1.42, 95%CI: 0.87–2.33), n-3 PUFA (HR: 1.16, 95%CI: 0.73–1.84), and n-6 PUFA (HR: 1.16, 95%CI: 0.69–1.93) were not significantly associated with the incidence of AML/MDS. Additionally, associations between the intake of interest and the incidence of AML/MDS did not show evidence of heterogeneity by age and sex, except for heterogeneity of small fish intake and sex (P for interaction: 0.048) (Supplemental Table 1).

**Table 2:** Acute myeloid leukemia and myelodysplastic syndrome risk by meat, fish and fatty acids intakes

	**Tertile of energy-adjusted food intake**			

	**Tertile 1**	**Tertile 2**	**Tertile 3**	**P_trend_^d^**
**Total meat**				
Median intake, g/day	22.8 (<36.6)	49.8 (36.6–65.4)	89.0 (>65.4)	
Person-years, year	444386	449493	451123	
Cases, n	41	32	43	
HR (95%CI)^a^	1.00 (reference)	0.89 (0.56–1.42)	1.33 (0.85–2.07)	0.224
HR (95%CI)^b^	1.00 (reference)	0.89 (0.56–1.42)	1.33 (0.85–2.08)	0.225
HR (95%CI)^c^	1.00 (reference)	1.02 (0.63–1.65)	1.40 (0.87–2.24)	0.169

**Red meat**				
Median intake, g/day	17.8 (<29.7)	41.3 (21.7–55.1)	76.4 (>55.1)	
Person-years, year	444781	449404	450816	
Cases, n	39	36	41	
HR (95%CI)^a^	1.00 (reference)	1.07 (0.68–1.68)	1.35 (0.85–2.13)	0.209
HR (95%CI)^b^	1.00 (reference)	1.07 (0.68–1.69)	1.35 (0.85–2.14)	0.208
HR (95%CI)^c^	1.00 (reference)	1.17 (0.73–1.88)	1.3 (0.85–2.25)	0.187

**Processed red meat**				
Median intake, g/day	0.5 (<2.1)	3.7 (2.1–6.1)	11.1 (>6.1)	
Person-years, year	440133	448860	456008	
Cases, n	40	32	44	
HR (95%CI)^a^	1.00 (reference)	1.00 (0.63–1.60)	1.61 (1.02–2.54)	0.044
HR (95%CI)^b^	1.00 (reference)	1.01 (0.63–1.61)	1.63 (1.03–2.57)	0.041
HR (95%CI)^c^	1.00 (reference)	1.09 (0.67–1.78)	1.67 (1.04–2.70)	0.038

**Unprocessed red meat**				
Median intake, g/day	14.8 (<25.2)	35.4 (25.2–47.7)	67.1 (>47.7)	
Person-years, year	445439	449569	449993	
Cases, n	41	38	37	
HR (95%CI)^a^	1.00 (reference)	1.03 (0.66–1.61)	1.08 (0.68–1.71)	0.744
HR (95%CI)^b^	1.00 (reference)	1.04 (0.67–1.62)	1.08 (0.68–1.71)	0.747
HR (95%CI)^c^	1.00 (reference)	1.10 (0.69–1.74)	1.12 (0.69–1.81)	0.638

**Poultry**				
Median intake, g/day	1.2 (<4.2)	6.5 (4.2–9.5)	14.6 (>9.5)	
Person-years, year	442015	450843	452143	
Cases, n	34	40	42	
HR (95%CI)^a^	1.00 (reference)	1.22 (0.77–1.93)	1.34 (0.85–2.12)	0.211
HR (95%CI)^b^	1.00 (reference)	1.22 (0.77–1.93)	1.35 (0.85–2.13)	0.207
HR (95%CI)^c^	1.00 (reference)	1.33 (0.82–2.17)	1.47 (0.90–2.38)	0.125

**Fish**				
Median intake, g/day	40.4 (<58.9)	76.2 (58.9–97.0)	127.8 (>97.0)	
Person-years, year	444689	449074	451238	
Cases, n	37	35	44	
HR (95%CI)^a^	1.00 (reference)	0.97 (0.60–1.55)	1.13 (0.72–1.79)	0.584
HR (95%CI)^b^	1.00 (reference)	0.97 (0.61–1.56)	1.13 (0.71–1.79)	0.586
HR (95%CI)^c^	1.00 (reference)	0.99 (0.60–1.62)	1.19 (0.74–1.93)	0.454

**Big Fish**				
Median intake, g/day	6.3 (<11.6)	17.0 (11.6–24.2)	35.9 (>24.2)	
Person-years, year	435909	451396	457696	
Cases, n	39	38	39	
HR (95%CI)^a^	1.00 (reference)	1.07 (0.67–1.73)	1.03 (0.64–1.67)	0.914
HR (95%CI)^b^	1.00 (reference)	1.09 (0.67–1.75)	1.03 (0.64–1.67)	0.917
HR (95%CI)^c^	1.00 (reference)	1.19 (0.71–1.97)	1.23 (0.74–2.05)	0.428

**Small Fish**				
Median intake, g/day	6.8 (<11.4)	16.3 (11.4–24.0)	37.4 (>24.0)	
Person-years, year	450314	448042	446644	
Cases, n	32	39	45	
HR (95%CI)^a^	1.00 (reference)	1.27 (0.79–2.05)	1.32 (0.81–2.13)	0.277
HR (95%CI)^b^	1.00 (reference)	1.26 (0.78–2.04)	1.32 (0.82–2.14)	0.269
HR (95%CI)^c^	1.00 (reference)	1.34 (0.81–2.22)	1.37 (0.83–2.28)	0.454

**Total FAs**				
Median intake, g/day	33.0 (<40.6)	46.6 (40.6–52.7)	60.2 (>52.7)	
Person-years, year	444236	450036	450728	
Cases, n	43	35	38	
HR (95%CI)^a^	1.00 (reference)	0.98 (0.62–1.54)	1.29 (0.81–2.06)	0.307
HR (95%CI)^b^	1.00 (reference)	1.00 (0.63–1.57)	1.31 (0.81–1.57)	0.289
HR (95%CI)^c^	1.00 (reference)	1.05 (0.65–1.68)	1.38 (0.83–2.28)	0.225

**Saturated FAs**				
Median intake, g/day	10.5 (<13.4)	13.4 (15.9–18.5)	22.0 (>18.5)	
Person-years, year	446481	450463	448057	
Cases, n	47	36	33	
HR (95%CI)^a^	1.00 (reference)	0.91 (0.59–1.42)	1.01 (0.63–1.61)	0.978
HR (95%CI)^b^	1.00 (reference)	0.92 (0.59–1.43)	1.01 (0.62–1.63)	0.984
HR (95%CI)^c^	1.00 (reference)	0.88 (0.55–1.39)	0.93 (0.56–1.55)	0.746

**MUFAs**				
Median intake, g/day	12.6 (<15.7)	18.2 (15.7–20.7)	24.1 (>20.7)	
Person-years, year	444418	449733	450850	
Cases, n	43	42	31	
HR (95%CI)^a^	1.00 (reference)	1.18 (0.76–1.81)	1.02 (0.63–1.67)	0.867
HR (95%CI)^b^	1.00 (reference)	1.18 (0.76–1.83)	1.03 (0.63–1.69)	0.858
HR (95%CI)^c^	1.00 (reference)	1.2 (0.78–1.93)	1.09 (0.65–1.83)	0.683

**PUFAs**				
Median intake, g/day	8.7 (<10.5)	12.0 (10.5–13.5)	15.4 (>13.5)	
Person-years, year	438631	449622	456748	
Cases, n	34	43	39	
HR (95%CI)^a^	1.00 (reference)	1.42 (0.90–2.24)	1.39 (0.86–2.26)	0.176
HR (95%CI)^b^	1.00 (reference)	1.45 (0.92–2.29)	1.42 (0.87–2.33)	0.157
HR (95%CI)^c^	1.00 (reference)	1.51 (0.93–2.46)	1.63 (0.98–2.73)	0.060

**n-3 PUFA**				
Median intake, g/day	1.6 (<2.0)	2.3 (2.0–2.7)	3.2 (>2.7)	
Person-years, year	441029	449351	453621	
Cases, n	37	38	41	
HR (95%CI)^a^	1.00 (reference)	1.10 (0.69–1.73)	1.15 (0.72–1.82)	0.562
HR (95%CI)^b^	1.00 (reference)	1.11 (0.70–1.75)	1.16 (0.73–1.84)	0.538
HR (95%CI)^c^	1.00 (reference)	1.15 (0.70–1.87)	1.35 (0.83–2.19)	0.223

**n-6 PUFA**				
Median intake, g/day	7.0 (<8.4)	9.5 (8.4–10.7)	12.3 (>10.7)	
Person-years, year	438156	449894	456951	
Cases, n	36	47	33	
HR (95%CI)^a^	1.00 (reference)	1.49 (0.96–2.31)	1.14 (0.69–1.88)	0.562
HR (95%CI)^b^	1.00 (reference)	1.51 (0.97–2.36)	1.16 (0.69–1.93)	0.527
HR (95%CI)^c^	1.00 (reference)	1.58 (0.99–2.53)	1.32 (0.78–2.24)	0.277

CI; confidence interval, HR; hazard ratio, FA: fatty acid, MUFA; monounsaturated fatty acid, and PUFA; polyunsaturated fatty acid

^a^Adjusted for age (continuous), sex, and study area (10 PHC areas).

^b^Adjusted for age (continuous), sex, study area (10 PHC area), body mass index (<23, 23–<25, 25–<27, and ≥27 kg/m^2^), history of smoking (no, past or current, and unknown), alcohol consumption frequency (never, rarely, 1–3 times/month, 1–2 times/week, 3–4 times/week, >4 times/week, and unknown); physical activity by metabolic equivalents/day (quartiles and unknown).

^c^Adjusted for the same covariates as in multivariable model 2 after excluding participants who were diagnosed with AML/MDS in the first 2 years to remove the potential bias of having AML/MDS at the start of the study.

^d^P_trend_ was used to test for a linear trends across tertiles as rank variables.

AML, acute myeloid leukemia; MDS, myelodysplastic syndromes

The HRs, 95% CIs, and P_trend_ of AML were shown in Table 3. None of the dietary factors of interest was significantly associated with the incidence of AML (Table 3). Among them, total meat (HR: 1.54, 95%CI: 0.88–2.69), red meat (HR: 1.74, 95%CI: 0.97–3.12), processed red meat (HR: 1.58, 95%CI: 0.89–2.82), and total fatty acids (HR: 1.77, 95%CI: 0.93–3.36) tend to be associated with an increased risk of AML.

**Table 3:** Acute myeloid leukemia risk by meat, fish and fatty acids intakes

	**Tertile of energy adjusted intake of interests**			

	**Tertile 1**	**Tertile 2**	**Tertile 3**	**P_trend_^b^**
**Total meat**				
Person year. year	444386	449493	451123	
Case, n	24	15	28	
HR (95%CI)^a^	1.00 (reference)	0.71 (0.37–1.36)	1.54 (0.88–2.69)	0.141
**Red meat**				
Person year. year	444781	449404	450816	
Case, n	21	19	27	
HR (95%CI)^a^	1.00 (reference)	1.05 (0.56–1.97)	1.74 (0.97–3.12)	0.067
**Processed red meat**				
Person year. year	440133	448860	456008	
Case, n	24	17	26	
HR (95%CI)^a^	1.00 (reference)	0.87 (0.46–1.63)	1.58 (0.89–2.82)	0.130
**Unprocessed red meat**				
Person year. year	445439	449569	449993	
Case, n	21	23	23	
HR (95%CI)^a^	1.00 (reference)	1.25 (0.69–2.27)	1.38 (0.76–2.53)	0.291
**Poultry**				
Person year. year	442015	450843	452143	
Case, n	19	24	24	
HR (95%CI)^a^	1.00 (reference)	1.33 (0.72–2.44)	1.35 (0.73–2.50)	0.342
**Fish**				
Person year. year	444689	449074	451238	
Case, n	18	21	28	
HR (95%CI)^a^	1.00 (reference)	1.11 (0.58–2.10)	1.33 (0.72–2.46)	0.349
**Big Fish**				
Person year. year	435909	451394	457696	
Case, n	21	23	23	
HR (95%CI)^a^	1.00 (reference)	1.05 (0.56–1.95)	0.91 (0.48–1.69)	0.931
**Small Fish**				
Person year. year	450314	448043	446644	
Case, n	17	25	25	
HR (95%CI)^a^	1.00 (reference)	1.47 (0.78–2.76)	1.37 (0.72–2.63)	0.367
**Total FAs**				
Person year. year	444236	450036	450728	
Case, n	21	22	24	
HR (95%CI)^a^	1.00 (reference)	1.30 (0.70–2.41)	1.77 (0.93–3.36)	0.082
**Saturated FAs**				
Person year. year	446481	450463	448057	
Case, n	23	22	22	
HR (95%CI)^a^	1.00 (reference)	1.18 (0.65–2.15)	1.48 (0.79–2.79)	0.224
**MUFAs**				
Person year. year	444418	449733	450850	
Case, n	21	27	19	
HR (95%CI)^a^	1.00 (reference)	1.57 (0.87–2.82)	1.35 (0.69–2.61)	0.343
**PUFAs**				
Person year. year	438631	449622	456748	
Case, n	20	24	23	
HR (95%CI)^a^	1.00 (reference)	1.35 (0.73–2.47)	1.35 (0.71–2.58)	0.363
**n-3 PUFA**				
Person year. year	441029	449351	453621	
Case, n	20	22	25	
HR (95%CI)^a^	1.00 (reference)	1.13 (0.61–2.10)	1.20 (0.65–2.22)	0.569
**n-6 PUFA**				
Person year. year	438156	449894	456951	
Case, n	20	24	23	
HR (95%CI)^a^	1.00 (reference)	1.37 (0.74–2.52)	1.42 (0.74–2.73)	0.287

CI; confidence interval, HR; hazard ratio, FA: fatty acid, MUFA; monounsaturated fatty acid, and PUFA; polyunsaturated fatty acid

^a^Adjusting for age (continuous), sex, study area (10 public health centers), body mass index (<23, 23–<25, 25–<27, and ≥27 kg/m^2^), history of smoking (no, past or current, and unknown), alcohol consumption frequency (never, rarely, 1–3 times/month, 1–2 times/week, 3–4 times/week, >4 times/week, and unknown); physical activity as metabolic equivalents/day (quartiles and unknown).

^b^P_trend_ was used to test for linear trends across tertiles as rank variables.

The HR, 95% CI, and P_trend_ of MDS were shown in Table 4. None of the dietary factors of interest was significantly associated with the incidence of MDS (Table 4). In contrast to AML, only processed red meat intake tended to associate with the incidence of MDS (HR: 1.66, 95%CI: 0.80–3.47).

**Table 4:** Myelodysplastic syndrome risk by meat, fish and fatty acids intakes

	**Tertile of energy-adjusted intake of interest**			

	**Tertile 1**	**Tertile 2**	**Tertile 3**	**P_trend_^b^**
**Total meat**				
Person year. year	444386	449492	451123	
Case, n	17	17	15	
HR (95%CI)^a^	1.00 (reference)	1.14 (0.58–2.24)	1.02 (0.49–2.14)	0.934
**Red meat**				
Person year. year	444781	449404	450816	
Case, n	18	17	14	
HR (95%CI)^a^	1.00 (reference)	1.09 (0.58–2.12)	0.88 (0.41–1.86)	0.764
**Processed red meat**				
Person year. year	440133	448860	456008	
Case, n	16	15	18	
HR (95%CI)^a^	1.00 (reference)	1.23 (0.60–2.51)	1.66 (0.80–3.47)	0.179
**Unprocessed red meat**				
Person year. year	445439	449569	449993	
Case, n	20	15	14	
HR (95%CI)^a^	1.00 (reference)	0.81 (0.41–1.59)	0.75 (0.36–1.55)	0.422
**Poultry**				
Person year. year	442015	450843	452143	
Case, n	15	16	18	
HR (95%CI)^a^	1.00 (reference)	1.09 (0.53–2.21)	1.33 (0.66–2.66)	0.422
**Fish**				
Person year. year	444689	449074	451238	
Case, n	19	14	16	
HR (95%CI)^a^	1.00 (reference)	0.83 (0.41–1.69)	0.91 (0.45–1.84)	0.778
**Big Fish**				
Person year. year	435909	451394	457696	
Case, n	18	15	16	
HR (95%CI)^a^	1.00 (reference)	1.16 (0.55–2.44)	1.25 (0.59–2.65)	0.569
**Small Fish**				
Person year. year	450314	448043	446644	
Case, n	15	14	20	
HR (95%CI)^a^	1.00 (reference)	1.02 (0.48–2.16)	1.25 (0.60–2.59)	0.541
**Total FAs**				
Person year. year	444236	450036	450729	
Case, n	22	13	14	
HR (95%CI)^a^	1.00 (reference)	0.71 (0.35–1.43)	0.87 (0.41–1.81)	0.631
**Saturated FAs**				
Person year. year	446481	450463	448057	
Case, n	24	14	11	
HR (95%CI)^a^	1.00 (reference)	0.66 (0.34–1.30)	0.57 (0.26–1.23)	0.128
**MUFAs**				
Person year. year	444418	4499733	450850	
Case, n	22	15	12	
HR (95%CI)^a^	1.00 (reference)	0.81 (0.42–1.60)	0.71 (0.33–1.52)	0.366
**PUFAs**				
Person year. year	438631	449622	456748	
Case, n	14	19	16	
HR (95%CI)^a^	1.00 (reference)	1.59 (0.79–3.21)	1.49 (0.70–3.20)	0.294
**n-3 PUFA**				
Person year. year	441029	449351	454621	
Case, n	17	16	16	
HR (95%CI)^a^	1.00 (reference)	1.07 (0.54–2.14)	1.10 (0.54–2.23)	0.796
**n-6 PUFA**				
Person year. year	438156	449894	456951	
Case, n	16	23	10	
HR (95%CI)^a^	1.00 (reference)	1.69 (0.88–3.25)	0.79 (0.34–1.84)	0.752

CI; confidence interval, HR; hazard ratio, FA: fatty acid, MUFA; monounsaturated fatty acid, and PUFA; polyunsaturated fatty acid

^a^Adjusting for age (continuous), sex, study area (10 public health centers), body mass index (<23, 23–<25, 25–<27, and ≥27 kg/m^2^), history of smoking (no, past or current, and unknown), alcohol consumption frequency (never, rarely, 1–3 times/month, 1–2 times/week, 3–4 times/week, >4 times/week, and unknown); physical activity as metabolic equivalents/day (quartiles and unknown).

^b^P_trend_ was used to test for linear trends across tertiles as rank variables.

## Discussion

Here we investigated the association between meat, fish, or fatty acid intake and the incidence of AML/MDS. Our results showed that a higher processed red meat intake was associated with an increased incidence of AML/MDS. On the other hand, other intakes of interest had a null association with the incidence of AML/MDS. Since there is little evidence regarding the association between meat, fish, or fatty acid intake and the incidence of AML/MDS, our results provide additional information regarding the etiology of AML and MDS.

Red meat and processed red meat are reportedly associated with an increased risk of several cancers through the development of carcinogens [11]. However, little epidemiological evidence is available regarding the association between meat intakes and the incidence of AML/MDS because of the limited number of studies, low incidence of AML/MDS, and differing definitions of leukemia, although leukemia subtypes have different etiologies. A cohort study from Europe revealed the null association between red meat or processed red meat and incidence of AML [24]. Similarly, two cohort studies from the United States revealed the null association between red meat or processed red meat and incidence of AML [22, 23]. For MDS, there have been even fewer epidemiological studies. A case-control study revealed that an increased intake of meat was associated with an increased incidence of MDS; however, a cohort study from the United States revealed a null association [25, 28]. The present study revealed that an increased intake of processed red meat was associated with an increased incidence of AML/MDS. Subtype analysis showed similar results, although the statistical significance was not maintained. Our results were inconsistent with those of previous studies conducted in the United States or Europe. A possible reason of the inconsistent result was that the intake of red meat and processed meat in Western countries were higher than that in Japan [40, 41]. The differing amounts of processed red meat intakes, which was smaller in our study than in previous studies, could have influenced the results if a smaller amount of processed red meat intake was associated with a decreased incidence of AML/MDS [22–24]. The chemicals contained in processed red meat such as antioxidants and preservatives could be associated with the incidence of AML/MDS [42]. Other possibilities were the difference in the geographic distribution of AML subtypes between the West and Asia and unmeasured confounders, such as professional exposure to chemicals. On the other hand, the intakes of saturated fatty acids, which is one of the component of meat, was not associated with the incidence of AML among epidemiological data, although the number of studies was limited and most of the studies were case-control in design [18, 26, 27]. Our results revealed that saturated fatty acid intake was not associated with the incidence of AML/MDS with HR of 1.01–1.42, a finding that was consistent with those of previous studies [18, 26, 27]. Further research is needed to assess the detailed etiology of the association between processed red meat intakes and the incidence of AML/MDS.

A recent meta-analysis that combined two cohort studies revealed that fish intake might increase the risk of the development of AML with an HR of 1.73 (95% CI, 1.22–2.47) for the highest versus lowest intake group [12], although fish are generally associated with reduced cancer risks via suppressing mutations, enhancing cell apoptosis, and inhibiting cell growth [43–45]. The results of the meta-analysis are considered due to fish containing some carcinogens such as sea pollution and dioxins that are related to AML development [12]. One of the European study included in the meta-analysis revealed that the median fish intake estimated from FFQ was 21.5 g/day, which was lower than our study and fish intake had trend to increase risk of AML with an HR of 1.50 (0.99–2.26) [24]. In contrast with the meta-analysis, our results indicated a null association between total fish, big and small fish intake and AML/MDS incidence, including subtype analysis. The different results from some previous reports might be due to the difference in amounts of fish intakes, the types of fish considering the small fish intake trend to higher risk of AML/MDS in our study, differences in environmental pollution of producing area, and eating habits of fish, and differences in AML/MDS subtypes.

Our study has some limitations. First, the effects of meat cooking methods, doneness levels, and additives were not considered due to a lack of data. Therefore, we have no suggestions or speculations regarding possible carcinogens such as heterocyclic amine, nitrites and so on [46, 47]. Second, it was unable to account for changes in exposure over the course of study period in this study. Third, unmeasured confounding factors may have influenced the intake of interest. Finally, the statistical power for the analysis might not be sufficient for the total outcome, especially subtypes. In addition, a detailed subtype analysis was not performed because of the insufficient incidence of AML. Therefore, larger studies and meta-analysis are required to confirm the results of this study. Readers should interpret our results cautiously while bearing these limitations in mind.

## Conclusion

We revealed that processed red meat was associated with an increased incidence of AML/MDS in the Japanese population.

## Abbreviations

AML: acute myeloid leukemia
BMI: body mass index
CI: confidence interval
FFQ: food frequency questionnaire
JPHC: Japan public health center
MDS: myelodysplastic syndrome
HR: hazard ratio

## Supplementary information

**Additional file 1:** **Supplemental Table 1.** Acute myeloid leukemia and myelodysplastic syndrome risk by meat, fish, and fatty acids intakes stratified by age and sex.

## Declarations

### Availability of data and materials

For information on how to gain access to JPHC data, follow the instructions at https://epi.ncc.go.jp/en/jphc/805/8155.html.
