# Supplementary material for: Association between meat, fish, and fatty acid intake and incidence of acute myeloid leukemia and myelodysplastic syndrome: the Japan Public Health Center-based Prospective Study
Source: Environ Health Prev Med. 2023 Mar 7;28:19. doi: 10.1265/ehpm.22-00233 (PMC10025862; doi:10.1265/ehpm.22-00233)
Supplement: Supplementary file 1 — Additional file 1: Supplemental Table 1. Acute myeloid leukemia and myelodysplastic syndrome risk by meat, fish, and fatty acids intakes stratified by age and sex. [file ehpm-28-019-s001.docx]

**Supplemental Materials**

Supplemental Table 1. Acute myeloid leukemia and myelodysplastic syndrome risk by meat, fish, and fatty acids intakes stratified by age and sex.

|  |  | Tertile of energy-adjusted food intake | | |  |
| --- | --- | --- | --- | --- | --- |
|  |  | Tertile 1 | Tertile 2 | Tertile 3 |  |
|  |  | Hazard ratio^1^ | Hazard ratio^1^ | Hazard ratio^1^ | P for interaction^2^ |
| **Total meat** |  |  |  |  |  |
| Age | Younger (-57) | 1.00 (Reference) | 1.36 (0.58-3.20) | 1.55 (0.66-3.66) | 0.519 |
|  | Elder (>57) | 1.00 (Reference) | 0.75 (0.42-1.32) | 1.32 (0.78-2.23) |  |
| Sex | Male | 1.00 (Reference) | 1.05 (0.59-1.88) | 1.41 (0.79-2.52) | 0.535 |
|  | Female | 1.00 (Reference) | 0.66 (0.30-1.45) | 1.20 (0.60-2.40) |  |
| **Red meat** |  |  |  |  |  |
| Age | Younger (-57) | 1.00 (Reference) | 1.67 (0.70-3.99) | 1.75 (0.71-4.29) | 0.500 |
|  | Elder (>57) | 1.00 (Reference) | 0.91 (0.52-1.57) | 1.28 (0.74-2.21) |  |
| Sex | Male | 1.00 (Reference) | 1.38 (0.77-2.47) | 1.62 (0.89-2.98) | 0.225 |
|  | Female | 1.00 (Reference) | 0.70 (0.33-1.49) | 1.03 (0.51-2.08) |  |
| **Processed meat** |  |  |  |  |  |
| Age | Younger (-57) | 1.00 (Reference) | 0.76 (0.31-1.84) | 1.36 (0.61-3.03) | 0.751 |
|  | Elder (>57) | 1.00 (Reference) | 1.14 (0.66-1.99) | 1.81 (1.04-3.14) |  |
| Sex | Male | 1.00 (Reference) | 1.11 (0.63-1.95) | 1.17 (0.64-2.14) | 0.207 |
|  | Female | 1.00 (Reference) | 0.81 (0.34-1.91) | 2.51 (1.22-5.16) |  |
| **Un-processed red meat** |  |  |  |  |  |
| Age | Younger (-57) | 1.00 (Reference) | 1.32 (0.59-2.99) | 1.20 (0.51-2.81) | 0.783 |
|  | Elder (>57) | 1.00 (Reference) | 0.94 (0.55-1.61) | 1.07 (0.62-1.85) |  |
| Sex | Male | 1.00 (Reference) | 1.10 (0.62-1.94) | 1.20 (0.67-2.16) | 0.597 |
|  | Female | 1.00 (Reference) | 0.94 (0.46-1.92) | 0.91 (0.43-1.89) |  |
| **Poultry** |  |  |  |  |  |
| Age | Younger (-57) | 1.00 (Reference) | 1.11 (0.48-2.58) | 1.45 (0.64-3.26) | 0.895 |
|  | Elder (>57) | 1.00 (Reference) | 1.28 (0.74-2.22) | 1.32 (0.75-2.30) |  |
| Sex | Male | 1.00 (Reference) | 1.07 (0.59-1.94) | 1.49 (0.84-2.63) | 0.381 |
|  | Female | 1.00 (Reference) | 1.52 (0.73-3.19) | 1.15 (0.53-2.52) |  |
| **Fish** |  |  |  |  |  |
| Age | Younger (-57) | 1.00 (Reference) | 0.90 (0.40-2.06) | 1.15 (0.50-2.62) | 0.930 |
|  | Elder (>57) | 1.00 (Reference) | 0.98 (0.55-1.75) | 1.08 (0.62-1.88) |  |
| Sex | Male | 1.00 (Reference) | 0.90 (0.51-1.61) | 1.10 (0.62-1.95) | 0.762 |
|  | Female | 1.00 (Reference) | 1.15 (0.50-2.64) | 1.21 (0.55-2.67) |  |
| **Big fish** |  |  |  |  |  |
| Age | Younger (-57) | 1.00 (Reference) | 0.72 (0.31-1.67) | 0.91 (0.41-2.00) | 0.371 |
|  | Elder (>57) | 1.00 (Reference) | 1.45 (0.80-2.62) | 1.20 (0.64-2.25) |  |
| Sex | Male | 1.00 (Reference) | 0.99 (0.54-1.81) | 1.05 (0.58-1.90) | 0.898 |
|  | Female | 1.00 (Reference) | 1.24 (0.55-2.78) | 1.09 (0.48-2.50) |  |
| **Small fish** |  |  |  |  |  |
| Age | Younger (-57) | 1.00 (Reference) | 1.53 (0.66-3.56) | 1.61 (0.68-3.85) | 0.705 |
|  | Elder (>57) | 1.00 (Reference) | 1.11 (0.63-1.95) | 1.07 (0.60-1.92) |  |
| Sex | Male | 1.00 (Reference) | 1.25 (0.68-2.30) | 1.32 (0.71-2.43) | 0.048 |
|  | Female | 1.00 (Reference) | 1.42 (0.67-2.99) | 0.90 (0.40-2.00) |  |
| **Total fatty acid** |  |  |  |  |  |
| Age | Younger (-57) | 1.00 (Reference) | 0.72 (0.31-1.65) | 0.93 (0.40-2.20) | 0.877 |
|  | Elder (>57) | 1.00 (Reference) | 1.14 (0.65-1.98) | 1.54 (0.86-2.75) |  |
| Sex | Male | 1.00 (Reference) | 0.77 (0.43-1.40) | 1.44 (0.79-2.62) | 0.173 |
|  | Female | 1.00 (Reference) | 1.42 (0.63-3.18) | 1.29 (0.56-2.96) |  |
| **Saturated fatty acid** |  |  |  |  |  |
| Age | Younger (-57) | 1.00 (Reference) | 0.83 (0.37-1.87) | 0.97 (0.41-2.28) | 0.834 |
|  | Elder (>57) | 1.00 (Reference) | 0.96 (0.56-1.63) | 1.03 (0.57-1.85) |  |
| Sex | Male | 1.00 (Reference) | 0.89 (0.51-1.55) | 0.93 (0.49-1.77) | 0.993 |
|  | Female | 1.00 (Reference) | 0.95 (0.44-2.04) | 1.11 (0.52-2.37) |  |
| **Monounsaturated fatty acid** |  |  |  |  |  |
| Age | Younger (-57) | 1.00 (Reference) | 0.92 (0.43-1.99) | 0.66 (0.27-1.62) | 0.841 |
|  | Elder (>57) | 1.00 (Reference) | 1.33 (0.78-2.26) | 1.27 (0.70-2.31) |  |
| Sex | Male | 1.00 (Reference) | 0.92 (0.52-1.61) | 1.20 (0.65-2.20) | 0.102 |
|  | Female | 1.00 (Reference) | 1.66 (0.78-3.53) | 0.91 (0.38-2.15) |  |
| **Poly-saturated fatty acid** |  |  |  |  |  |
| Age | Younger (-57) | 1.00 (Reference) | 0.89 (0.41-1.91) | 0.66 (0.27-1.60) | 0.382 |
|  | Elder (>57) | 1.00 (Reference) | 1.86 (1.03-3.33) | 2.01 (1.09-3.71) |  |
| Sex | Male | 1.00 (Reference) | 1.36 (0.77-2.40) | 1.89 (1.04-3.44) | 0.146 |
|  | Female | 1.00 (Reference) | 1.46 (0.65-3.25) | 0.95 (0.41-2.20) |  |
| **n-3 poly-saturated fatty acid** |  |  |  |  |  |
| Age | Younger (-57) | 1.00 (Reference) | 1.50 (0.70-3.22) | 0.92 (0.37-2.28) | 0.300 |
|  | Elder (>57) | 1.00 (Reference) | 0.91 (0.51-1.62) | 1.19 (0.69-2.05) |  |
| Sex | Male | 1.00 (Reference) | 0.89 (0.50-1.60) | 1.37 (0.79-2.39) | 0.171 |
|  | Female | 1.00 (Reference) | 1.56 (0.68-3.58) | 1.01 (0.43-2.37) |  |
| **n-6 poly-saturated fatty acid** |  |  |  |  |  |
| Age | Younger (-57) | 1.00 (Reference) | 0.59 (0.26-1.31) | 0.61 (0.26-1.44) | 0.060 |
|  | Elder (>57) | 1.00 (Reference) | 2.37 (1.35-4.17) | 1.63 (0.85-3.12) |  |
| Sex | Male | 1.00 (Reference) | 1.44 (0.84-2.48) | 1.35 (0.71-2.54) | 0.498 |
|  | Female | 1.00 (Reference) | 1.55 (0.70-3.44) | 0.96 (0.41-2.25) |  |

^1^Adjusted for covarites other than those stratified among the following; age (continuous), sex, study area (10 PHC area), body mass index (<23, 23– <25, 25– <27, and ≥27 kg/m^2^), history of smoking (no, past or current, and unknown), alcohol consumption frequency (never, rarely, 1–3 times/month, 1–2 times/week, 3–4 times/week, >4 times/week, and unknown); physical activity by metabolic equivalents/day (quartiles and unknown).

^2^P for trend was used to test for a linear trend across tertile as rank variables.
